# Supplementary material for: What are the prognostic factors for the development of incontinence-associated dermatitis (IAD): a protocol for a systematic review and meta-analysis
Source: BMJ Open. 2023 Jul 10;13(7):e073115. doi: 10.1136/bmjopen-2023-073115 (PMC10335443; doi:10.1136/bmjopen-2023-073115)
Supplement: Supplementary data [file bmjopen-2023-073115supp001.pdf]

Field codes:
/= MeSH term
exp= exploded MeSH term
ab.= abstract
kf.= keyword heading word
ti.= title
tw.= title/abstract

Database(s): **Ovid MEDLINE(R) ALL** 1946 to April 13, 2023
Search Strategy:

#	Searches
**Incontinence**	
1	exp Urinary Incontinence/
2	Incontinence Pads/
3	Absorbent Pads/
4	Diapers, Adult/
5	Fecal Incontinence/
6	(incontinen* or diaper* or napkin* or nappy or perineal or perineum or "absorbent pads" or underpad*).ab,kf,ti.
7	1 or 2 or 3 or 4 or 5 or 6
**Dermatis/skin problems**	
8	Dermatitis/ or exp Dermatitis, Contact/ or exp Intertrigo/ or exp Dermatitis, Irritant/ or "Erythema"/
9	(dermati* or erythema or rash* or intertrig* or erythema or "moisture associated skin damage" or MASD or "skin barrier*" or "skin breakdown" or "skin condition" or "skin damage" or "skin health" or "fragile skin" or "heathy skin" or "skin problem*" or erythema or "perianal skin" or "Buttock skin" or "wetness lesion*" or "moisture lesion*").ab,kf,ti.
10	8 or 9
**Prognostic factors**	
11	exp Risk/
12	risk.tw.
13	exp Cohort Studies/
14	cohort.tw.
15	exp Prognosis/
16	"prognos*".tw.
17	"predict*".tw.
18	exp Incidence/
19	incidence.tw.
20	"causal factor".tw.

21	course.tw.
22	exp Follow-Up Studies/
23	(prognos* or predict* or risk or incidence or indicat* or factor* or predisposition or predisposed or etiology or aetiology).ab,kf,ti.
24	11 or 12 or 13 or 14 or 15 or 16 or 17 or 18 or 19 or 20 or 21 or 22 or 23
**Sets combined**	
25	7 and 10 and 24

Published search filters for prognosis/prognostic factors were consulted in the development of the search strategy. Line 11-21 can be found in the filter by Stallings et al.(1). Line 16, 17 and 21 are also used I by Kavanagh et al.(2). Both filters also use additional terms associated with mortality, but those terms were not used since IAD is not mortal. Because articles on prognostic factors may still use a terms not present in the filters, line 23 also includes some additional terms.

1. Stallings E, Gaetano-Gil A, Alvarez-Diaz N, Sola I, Lopez-Alcalde J, Molano D, et al. Development and evaluation of a search filter to identify prognostic factor studies in Ovid MEDLINE. Bmc Med Res Methodol. 2022;22:107. DOI: 10.1186/s12874-022-01595-9

2. Kavanagh PL, Frater F, Navarro T, LaVita P, Parrish R, Iorio A. Optimizing a literature surveillance strategy to retrieve sound overall prognosis and risk assessment model papers. J Am Med Inform Assoc JAMIA. 2021;28:766–71.
