# Supplementary material for: What are the prognostic factors for the development of incontinence-associated dermatitis (IAD): a protocol for a systematic review and meta-analysis
Source: BMJ Open. 2023 Jul 10;13(7):e073115. doi: 10.1136/bmjopen-2023-073115 (PMC10335443; doi:10.1136/bmjopen-2023-073115)
Supplement: Author's manuscript [file bmjopen-2023-073115.draft_revisions.pdf]

BMJ Open is committed to open peer review. As part of this commitment we make the peer review history of every article we publish publicly available.

When an article is published we post the peer reviewers' comments and the authors' responses online. We also post the versions of the paper that were used during peer review. These are the versions that the peer review comments apply to.

The versions of the paper that follow are the versions that were submitted during the peer review process. They are not the versions of record or the final published versions. They should not be cited or distributed as the published version of this manuscript.

BMJ Open is an open access journal and the full, final, typeset and author-corrected version of record of the manuscript is available on our site with no access controls, subscription charges or pay-per-view fees (http://bmjopen.bmj.com).

If you have any questions on BMJ Open's open peer review process please email info.bmjopen@bmj.com

BMJ Open

What are the prognostic factors for the development of incontinence-associated dermatitis (IAD): A protocol for a Systematic Review and Meta-Analysis

Journal:	*BMJ Open*
Manuscript ID	bmjopen-2023-073115
Article Type:	Protocol
Date Submitted by the Author:	23-Feb-2023

Complete List of Authors:	Deprez, Julie; Örebro universitet, Swedish Centre for Skin and Wound Research (SCENTR), School of Health Sciences; Ghent University, Skin Integrity Research Group (SKINT), University Centre for Nursing and Midwifery, Department of Public Health and Primary Care<br>Kottner, Jan; Charite Universitatsmedizin Berlin, Institute for Clinical Nursing Science; Ghent University, Skin Integrity Research Group (SKINT), University Centre for Nursing and Midwifery, Department of Public Health and Primary Care<br>Eilegård Wallin, Alexandra; Örebro universitet, Swedish Centre for Skin and Wound Research (SCENTR), School of Health Sciences<br>Ohde, Nils; Charite Universitatsmedizin Berlin, Institute for Clinical Nursing Science<br>Bååth, Carina ; Karlstad University, Department of Health Sciences, Faculty of Health, Science and Technology; Østfold University College - Campus Frederikstad, Faculty of Health, Welfare and Organisation<br>Hommel, Ami ; Malmo University, Department of Care Science<br>Hultin, Lisa; Upsala University , Department of Public Health and Caring Sciences; Upsala University Hospital<br>Josefson, Anna; Örebro universitet, School of Medical Sciences, Faculty of Medicine and Health; Örebro University Hospital, Department of Dermatology<br>Beeckman, Dimitri; Örebro universitet, Swedish Centre for Skin and Wound Research (SCENTR), School of Health Sciences; Ghent University, Skin Integrity Research Group (SKINT), University Centre for Nursing and Midwifery, Department of Public Health and Primary Care

Keywords:	Prognosis, Systematic Review, Risk Factors, WOUND MANAGEMENT

SCHOLARONE™
Manuscripts

TITLE PAGE

Article Title: *What are the prognostic factors for the development of incontinence-associated dermatitis (IAD): A protocol for a Systematic Review and Meta-Analysis*

Author Information:

DEPREZ Julie[1,2], KOTTNER Jan[2,3], EILEGÅRD WALLIN Alexandra[1], OHDE Nils[3], BÅÅTH Carina[4,5], HOMMEL, Ami[6], HULTIN Lisa[7,8], JOSEFSON Anna[9,10], BEECKMAN, Dimitri[1,2,11,12]

[1] Swedish Centre for Skin and Wound Research (SCENTR), Faculty of Health and Medicine, School of Health Sciences, Örebro University, Örebro, Sweden

[2] Skin Integrity Research Group (SKINT), University Centre for Nursing and Midwifery, Department of Public Health and Primary Care, Ghent University, Ghent, Belgium

[3] Institute for Clinical Nursing Science, Charité Universitätsmedizin Berlin, Berlin, Germany

[4] Department of Health Sciences, Faculty of Health, Science and Technology, Karlstad University, Karlstad, Sweden

[5] Faculty of Health, Welfare and Organisation, Østfold University College, Fredrikstad, Norway

[6] Department of Care Science Malmö University, Malmö University, Malmö, Sweden

[7] Department of Public Health and Caring Sciences, Uppsala University, Uppsala, Sweden

[8] Uppsala University Hospital, Uppsala, Sweden

[9] School of Medical Sciences, Faculty of Health and Medicine, Örebro University, Örebro, Sweden

[10] Department of Dermatology, University Hospital Örebro, Örebro, Sweden

[11] Research Unit of Plastic Surgery, Department of Clinical Research, Faculty of Health Sciences, Odense, Denmark

[12] School of Nursing & Midwifery, Royal College of Surgeons in Ireland (RCSI), Dublin, Ireland

CORRESPONDING AUTHOR

Julie DEPREZ
C. Heymanslaan 10, 9000 Ghent, Belgium
julie.deprez@oru.se

Word Count: 1.577

Keywords:
Prognostic factors, prognosis, risk factors, incontinence associated dermatitis, systematic review, meta-analysis.

For peer review only

ABSTRACT

Introduction

Incontinence associated dermatitis (IAD) is irritant contact dermatitis and skin damage associated with prolonged skin contact with urine and/or faeces. Identifying prognostic factors for the development of IAD may improve management, facilitate prevention, and inform future research.

Methods and analysis

This protocol follows the guidelines of the Preferred Reporting Items for Systematic Review and Meta-Analysis Protocols (PRISMA-P). Prospective observational studies or clinical trials in which prognostic factors associated with the development of IAD are described are eligible. There are no restrictions on study setting, time, language, participant characteristics, or geographic regions. Reviews, editorials, commentaries, methodological articles, letters to the editor, cross-sectional and case-control studies, and case reports, are excluded. PubMed, CINAHL, EMBASE, and The Cochrane Library will be searched. Two independent reviewers will independently evaluate studies. The Quality in Prognostic Studies tool will be used to assess the risk of bias, and the CHARMS-PF checklist will be used for data extraction of the included studies. Separate analyses will be conducted for each identified prognostic factor, with adjusted and unadjusted estimated measures analysed separately. Evidence will be summarised with a meta-analysis when possible, and narratively otherwise. The Q and I2 statistics will be calculated in order to quantify heterogeneity. The quality of the evidence obtained will be evaluated according to the Grades of Recommendation Assessment, Development and Evaluation guidance.

Ethics and dissemination

No ethical approval is needed since all data is already publicly accessible. The results of this work will be published in a peer-reviewed scientific journal.

ARTICLE SUMMARY

STRENGTHS AND LIMITATIONS OF THIS STUDY

- This is the first systematic review to summarize the quality of evidence of prognostic factors for IAD.
- The risk of bias and the certainty of evidence will be evaluated using state-of-the-art methods for each prognostic factor separately.
- Because of interaction and confounding the generalisability of the identified prognostic factors might be limited.

For peer review only

MAIN TEXT

INTRODUCTION

Incontinence associated dermatitis (IAD) is one form of irritant contact dermatitis (EK02.22) and describes cutaneous inflammation and skin damage associated with prolonged skin contact with urine and/or faeces. (1, 2)

Clinical signs of IAD include skin erythema and oedema, occasionally with bullae and serous exudate, erosion, or subsequent cutaneous infection. (3, 4) Patients with IAD may experience discomfort, pain, burning, itching, or tingling. (5) The presence of IAD can result in an excessive burden of care, loss of independence, disruption in activities and/or sleep, and a reduction in quality of life, which worsens with higher frequency and quantity of soiling. (1, 6, 7) Additionally, IAD is associated with an increased risk of secondary infections, and the development of pressure ulcers. (1, 8, 9, 10) Managing all of these require additional resources, including prolonged hospital stays, resulting in a rise in healthcare expenses. (4, 11)

Prevalence ranges between 5.2% and 46%, depending on the type of setting and population under study. (9, 12)

By definition, incontinence is a prerequisite for having IAD, but in fact not all incontinent individuals develop IAD. (13) This suggests that many other factors and characteristics increase or decrease a person's susceptibility of developing IAD. Risk factors for IAD have been described (14, 15), but to our knowledge a systematic review and evidence appraisal on prognostic factors has not yet been conducted. 'A prognostic factor is any variable that is associated with the risk of a subsequent health outcome among people with a particular health condition.' (16, p. 2)

As such, our objective is to conduct a systematic review, and meta-analysis of prognostic factors associated with the development of IAD.

Review question

What are prognostic factors associated with the development of IAD in incontinent patients?

METHODS AND ANALYSIS

This protocol conforms to the Preferred Reporting Items for Systematic Reviews and Meta-Analyses Protocols (PRISMA-P) checklist. The protocol will be registered with the PROSPERO international prospective register of systematic reviews.

Eligibility criteria

Table 1 shows the description of the PICOTS (Population, Index prognostic factor, Comparator prognostic factor, Outcome, Timing, Setting) aspects. (16) The following study designs are eligible: RCTs, CCTs, prospective and retrospective cohort studies. Reviews, editorials, commentaries, methodological articles, letters to the editor, cross-sectional studies, case-control studies, and case reports, are excluded. There are no restrictions on study setting, time, language, participant characteristics, or geographic regions. Due to the variety of language used to describe incontinence-

For peer review only - http://bmjopen.bmj.com/site/about/guidelines.xhtml

associated skin problems (17, 18), all variations of terms will be included. If they describe the skin's reactive response to repeated exposure to urine and/or faeces, which may appear as inflammation and erythema with or without erosion or denudation. (3, 4, 19)

TABLE 1: Key items for framing aim, search strategy, and study inclusion and exclusion criteria for systematic review, following PICOTS

	Definition
**Population**	Incontinent adults (>18y) and adolescents (age 10 – 18y) (20)<br>Excluded: New borns, infants and young children (<10y)
**Index prognostic factor**	Any variable collected prior to diagnosis of IAD and assessed for association with IAD
**Comparator prognostic factors**	Any other variable that was collected before the diagnosis of IAD
**Outcomes**	(1) Development of a new IAD<br>(2) Severity of IAD
**Time**	Minimum: 3 days<br>Maximum: the duration of the study or observation period
**Setting**	Any healthcare setting (Examples include but are not limited to: hospitals, nursing homes, community care…)

Information sources

To identify articles, the databases PubMed, CINAHL, EMBASE, and The Cochrane Library will be searched. Additional relevant studies will be identified by cited and citing references. ClinicalTrials.gov (www.clinicaltrials.gov) and the WHO International Clinical Trials Registry Platform (apps.who.int/trialsearch/) will be searched for unpublished and on-going studies. Articles will be electronically retrieved and in cases where electronic copies are unavailable, hard copies of these articles will be requested from the university library.

Search strategy

A library technician with expertise in systematic reviews at the Örebro University, Örebro, Sweden has been consulted to develop the search methods. The search strategy will consist of key terms combined with Boolean operators. Key terms will be defined using the PICOTS and will include incontinence associated dermatitis, IAD, and prognostic factors using various combinations and synonyms. The Boolean operator OR will be used to combine search terms for the same concept. The Boolean operator AND will be used to combine the concepts. The search strategy will be adapted to each database. When appropriate MeSH terms or comparable alternatives will be used.

Study records

Data management

For peer review only - http://bmjopen.bmj.com/site/about/guidelines.xhtml

Retrieved records will be imported in Endnote and duplicates will be removed using the duplicate search function and by manually reviewing the list. Articles will then be imported into Covidence (21), an online tool that facilitates blind article screening by multiple reviewers.

Selection process

Titles and abstracts of all identified records will be reviewed for suitability by two researchers independently using the systematic review software Covidence (21). For each excluded article, reasons for exclusion will be specified. Disagreement or doubt will be resolved by consensus and if consensus cannot be reached, a full-text screening will be conducted, and a third reviewer will be consulted. The full texts of the remaining articles will be individually assessed for eligibility by the same reviewers. Any doubts will be resolved by a third reviewer.

Data collection process

The data extraction will be independently completed by two reviewers. Data extraction forms will be used to extract the relevant information and evidence. Any disagreement will be resolved through consultation with another reviewer.

Data items

The Checklist for Critical Appraisal and Data Extraction for Systematic Reviews of Prediction Modelling Studies – Prognostic Factors (CHARMS-PF) (16) will guide data extraction. It has nine domains that cover information required to extract data in a reliable manner: source of data, participants, outcomes to be predicted, prognostic factors, sample size, missing data, analysis, results and interpretation and discussion. Table 2 shows a list of data items that will be extracted.

TABLE 2: Data charting variables and domains relating to article description

Domain	Selected items and examples
**General study information**	Title, authors names, year of publication
**Study characteristics**	Sample size, study design, duration of follow-up, geographical location
**Participant characteristics**	Participant description, eligibility criteria
**Setting**	Type of care institution (hospital (unit), homecare, residential care…)
**Definition of outcomes**	IAD definition used by authors.<br>Incontinence definition used by authors.
**Prognostic factors**	Examples include but are not limited to: presence of faecal incontinence and/or urinary incontinence, care dependency, level of personal hygiene…
**Missing data**	Attrition (quantification and reasons), handling of missing data by the study authors
**Analysis**	Univariate analysis/logistic regression/Cox regression/other

For peer review only - http://bmjopen.bmj.com/site/about/guidelines.xhtml

**Results**	*Estimates reported between the prognostic factor and each review outcome*: (a) unadjusted estimate (b) adjusted estimates. *Type of measure of association*: odds ratio (OR), risk ratio (RR) or hazard ratio (HR)

Outcomes and prioritization

The main predicted outcome will be the development of new IAD, defined as an irritant contact dermatitis resulting from prolonged contact of the skin with urine and/or faeces. (2) The severity of IAD will also be examined.

Assessment of a risk of bias in individual studies

The Quality in Prognostic Factor Studies (QUIPS) (22) tool will be used to assess the risk of bias of each of the prognostic factors in individual studies. It has six domains (study participants, study attrition, prognostic factor measurement, outcome measurement, study confounding and statistical analysis and reporting) with a series of prompting items for each domain. Each domain is assigned a risk of bias rating of high, moderate, or low, and the overall risk of bias of a study is determined by a cumulative rating of all domains. The risk of bias will be independently assessed by two reviewers. Disagreements will be discussed with a third author until consensus is reached.

Data synthesis

A table will be created summarizing the characteristics of identified prognostic factors. Then, the strength and directions of association will be summarized for each prognostic factor separately. ORs, RRs, and HRs will be used as measures of association. Mean differences will be considered for continuous prognostic factors. Adjusted and unadjusted estimates for each prognostic factor will be analysed separately. When at least five studies report similar measures of association, a meta-analysis will be conducted for each prognostic factor, if the measurement of this factor is comparable. (16, 23) When neither OR, RR, or HR nor corresponding SEs are reported, any available data from the included study, such as confidence intervals, Kaplan-Meier curves, logrank test p values, will be utilized to estimate the associations if possible. (24, 25) A random-effects model will be used when conducting meta-analysis, since it is assumed that there is a high degree of variability among studies due to clinical and methodological differences. (26) The effects of each prognostic factor will be estimated as an OR or standardised mean difference with a 95% CI and T2, which indicates between-study variance. Furthermore, a 95% prediction interval will also be calculated. (27) The heterogeneity between studies in each meta-analysis will be assessed visually by examining forest plots and calculating the Q and I2 statistics. (28) When it is not feasible to conduct a quantitative synthesis, the evidence will be summarised narratively.

Bias

The presence of small study bias, including publication bias, will be evaluated graphically by examining the presence of asymmetry in a funnel plots. Additionally, the Egger's test will be used to make a statistical assessment if a meta-analysis is based on 10 or more studies for an outcome. (29) For this test, the natural logarithmic scale of the OR must be regressed against its SE, and statistical significance for asymmetry is set at the 10% level due to the low power of the test. If publication bias is statistically suspected, the method of trim and fill can be used to estimate the number of missing studies and

For peer review only - http://bmjopen.bmj.com/site/about/guidelines.xhtml

provide an adjusted summary effect. (30) To assess selective reporting, the consistency of study findings with its protocol must be examined, if available.

Confidence in cumulative evidence

Using the modified GRADE approach, the quality of evidence for each prognostic factor will be evaluated. The certainty rating for observational studies will begin at high, while the secondary analysis of highly controlled RCTs will start at low. The domains of risk of bias, inconsistency, indirectness, imprecision, publication bias, large effect, dose response, and plausible confounding will be taken into account when deciding to downgrade or upgrade the evidence's certainty. (31)

Patient and public involvement

No patients or service users were involved in the design of this review protocol.

ETHICS AND DISSEMINATION

This systematic review does not require ethics approval as it will be a summary of previously published evidence. No individual patient data will be obtained or accessed. Therefore, there is no concerning ethical issue in the conduct of this research. Following the completion of the systematic review, findings will be presented to academic audiences at international conferences focusing on skin integrity. The results will also be published in a peer-reviewed academic journal to reach clinical and academic experts interested in the topic. Plain language summaries and presentations to hospitals and other relevant clinical programmes will also be developed.

For peer review only

For peer review only - http://bmjopen.bmj.com/site/about/guidelines.xhtml

FOOTNOTES

Author Contributions:

All authors have made substantial contributions to all of the following: (1) the conception and design of the protocol (2) drafting the protocol or revising it critically for important intellectual content, (3) final approval of the version to be submitted.

Conflict of interest statement:

The authors declare that there is no conflict of interest.

Acknowledgments:

We acknowledge the support and funding from Vetenskapsrådet.

Funding Statement:

This work was supported by Vetenskapsrådet, grant number 2021-02653.

Data Availability Statement:

Data sharing not applicable as no datasets generated and/or analysed for this study.

For peer review only

REFERENCES

1. Beeckman D, Campbell J, Campbell K, et al. Proceedings of the Global IAD Expert Panel. Incontinence associated dermatitis: moving prevention forward. Wounds International. 2015.
2. World Health Organization (WHO). International classification of diseases for mortality and morbidity statistics (11th Revision). 2018. Available from: https://icd.who.int/browse11/l-m/en [Accessed 9 Jan 2023].
3. Beeckman D, Van Damme N, Schoonhoven L, et al. Interventions for preventing and treating incontinence-associated dermatitis in adults. Cochrane Database Syst Rev. 2016;11(11):Cd011627.
4. Raepsaet C, Fourie A, Van Hecke A, et al. Management of incontinence-associated dermatitis: A systematic review of monetary data. International wound journal. 2021;18(1):79-94.
5. Van den Bussche K, Kottner J, Beele H, et al. Core outcome domains in incontinence-associated dermatitis research. Journal of advanced nursing. 2018;74(7):1605-17.
6. Bartlett L, Nowak M, Ho YH. Impact of fecal incontinence on quality of life. World J Gastroenterol. 2009;15(26):3276-82.
7. Minassian VA, Devore E, Hagan K, et al. Severity of urinary incontinence and effect on quality of life in women by incontinence type. Obstetrics and gynecology. 2013;121(5):1083-90.
8. Demarre L, Verhaeghe S, Van Hecke A, et al. Factors predicting the development of pressure ulcers in an at-risk population who receive standardized preventive care: secondary analyses of a multicentre randomised controlled trial. Journal of advanced nursing. 2015;71(2):391-403.
9. Gray M, Giuliano KK. Incontinence-Associated Dermatitis, Characteristics and Relationship to Pressure Injury: A Multisite Epidemiologic Analysis. J Wound Ostomy Continence Nurs. 2018;45(1):63-7.
10. Van Damme N, Vanryckeghem E, Verhaeghe S, et al. Incontinence-associated dermatitis in elderly : a qualitative phenomenological study on patient experiences. 18th Annual meeting of the European Pressure Ulcer Advisory Panel (EPUAP 2015); Ghent, Belgium. European Pressure Ulcer Advisory Panel, 18th Annual meeting, Abstracts: EPUAP Business Office; 2015. p. 65 -
11. Cunich M, Barakat-Johnson M, Lai M, et al. The costs, health outcomes and cost-effectiveness of interventions for the prevention and treatment of incontinence-associated dermatitis: A systematic review. Int J Nurs Stud. 2022;129:104216.
12. Babino G, Argenziano G. Incontinence-Associated Dermatitis: An Insidious and Painful Condition. In: Docimo L, Brusciano L, editors. Anal Incontinence: Clinical Management and Surgical Techniques. Cham: Springer International Publishing; 2023. p. 179-87.
13. Van Damme N, Clays E, Verhaeghe S, et al. Independent risk factors for the development of incontinence-associated dermatitis (category 2) in critically ill patients with fecal incontinence: A cross-sectional observational study in 48 ICU units. Int J Nurs Stud. 2018;81:30-9.
14. Heywood N, Holloway S. Risk factors for incontinence associated dermatitis: an evidence-based review. Wounds UK. 2014;10(4):22 - 33.
15. Kottner J, Blume-Peytavi U, Lohrmann C, et al. Associations between individual characteristics and incontinence-associated dermatitis: a secondary data analysis of a multi-centre prevalence study. Int J Nurs Stud. 2014;51(10):1373-80.

For peer review only

16. Riley RD, Moons KGM, Snell KIE, et al. A guide to systematic review and meta-analysis of prognostic factor studies. BMJ. 2019;364:k4597.
17. Beeckman D, Schoonhoven L, Verhaeghe S, et al. Prevention and treatment of incontinence-associated dermatitis: literature review. J Adv Nurs. 2009;65(6):1141-54.
18. Dunk AM, Broom M, Kottner J, et al. Is it time to reconsider the terminology for diaper dermatitis? J Tissue Viability. 2023.
19. Gray M, Bohacek L, Weir D, et al. Moisture vs pressure: making sense out of perineal wounds. J Wound Ostomy Continence Nurs. 2007;34(2):134-42.
20. World Health Organization (WHO). Adolescents Health. Available from: https://www.who.int/health-topics/adolescent-health#tab=tab_1 [Accessed 25 Jan 2023].
21. Veritas Health Innovation. Covidence systematic review software. 2023.
22. Hayden JA, van der Windt DA, Cartwright JL, et al. Assessing bias in studies of prognostic factors. Ann Intern Med. 2013;158(4):280-6.
23. Jackson D, Turner R. Power analysis for random-effects meta-analysis. Res Synth Methods. 2017;8(3):290-302.
24. Parmar MK, Torri V, Stewart L. Extracting summary statistics to perform meta-analyses of the published literature for survival endpoints. Stat Med. 1998;17(24):2815-34.
25. Tierney JF, Stewart LA, Ghersi D, et al. Practical methods for incorporating summary time-to-event data into meta-analysis. Trials. 2007;8:16.
26. DerSimonian R, Laird N. Meta-analysis in clinical trials. Control Clin Trials. 1986;7(3):177-88.
27. Riley RD, Higgins JPT, Deeks JJ. Interpretation of random effects meta-analyses. BMJ. 2011;342:d549.
28. Higgins JPT, Thompson SG, Deeks JJ, et al. Measuring inconsistency in meta-analyses. BMJ. 2003;327(7414):557-60.
29. Egger M, Smith GD, Schneider M, et al. Bias in meta-analysis detected by a simple, graphical test. BMJ. 1997;315(7109):629-34.
30. Duval S, Tweedie R. Trim and fill: A simple funnel-plot-based method of testing and adjusting for publication bias in meta-analysis. Biometrics. 2000;56(2):455-63.
31. Foroutan F, Guyatt G, Zuk V, et al. GRADE Guidelines 28: Use of GRADE for the assessment of evidence about prognostic factors: rating certainty in identification of groups of patients with different absolute risks. J Clin Epidemiol. 2020;121:62-70.

For peer review only

PRISMA-P (Preferred Reporting Items for Systematic review and Meta-Analysis Protocols) 2015 checklist: recommended items to address in a systematic review protocol*

Section and topic	Item No	Checklist item	Reported on page #
**ADMINISTRATIVE INFORMATION**			
Title:			
Identification	1a	Identify the report as a protocol of a systematic review	1
Update	1b	If the protocol is for an update of a previous systematic review, identify as such	n/a
Registration	2	If registered, provide the name of the registry (such as PROSPERO) and registration number	n/a
Authors:			
Contact	3a	Provide name, institutional affiliation, e-mail address of all protocol authors; provide physical mailing address of corresponding author	1
Contributions	3b	Describe contributions of protocol authors and identify the guarantor of the review	8
Amendments	4	If the protocol represents an amendment of a previously completed or published protocol, identify as such and list changes; otherwise, state plan for documenting important protocol amendments	n/a
Support:			
Sources	5a	Indicate sources of financial or other support for the review	8
Sponsor	5b	Provide name for the review funder and/or sponsor	8
Role of sponsor or funder	5c	Describe roles of funder(s), sponsor(s), and/or institution(s), if any, in developing the protocol	n/a
**INTRODUCTION**			
Rationale	6	Describe the rationale for the review in the context of what is already known	3
Objectives	7	Provide an explicit statement of the question(s) the review will address with reference to participants, interventions, comparators, and outcomes (PICO)	3
**METHODS**			
Eligibility criteria	8	Specify the study characteristics (such as PICO, study design, setting, time frame) and report characteristics (such as years considered, language, publication status) to be used as criteria for eligibility for the review	3 + 4
Information sources	9	Describe all intended information sources (such as electronic databases, contact with study authors, trial registers or other grey literature sources) with planned dates of coverage	4
Search strategy	10	Present draft of search strategy to be used for at least one electronic database, including planned limits, such that it could be repeated	n/a

For peer review only

Study records:			
Data management	11a	Describe the mechanism(s) that will be used to manage records and data throughout the review	4 + 5
Selection process	11b	State the process that will be used for selecting studies (such as two independent reviewers) through each phase of the review (that is, screening, eligibility and inclusion in meta-analysis)	5
Data collection process	11c	Describe planned method of extracting data from reports (such as piloting forms, done independently, in duplicate), any processes for obtaining and confirming data from investigators	5
Data items	12	List and define all variables for which data will be sought (such as PICO items, funding sources), any pre-planned data assumptions and simplifications	5 + 6
Outcomes and prioritization	13	List and define all outcomes for which data will be sought, including prioritization of main and additional outcomes, with rationale	6
Risk of bias in individual studies	14	Describe anticipated methods for assessing risk of bias of individual studies, including whether this will be done at the outcome or study level, or both; state how this information will be used in data synthesis	6
Data synthesis	15a	Describe criteria under which study data will be quantitatively synthesised	6
	15b	If data are appropriate for quantitative synthesis, describe planned summary measures, methods of handling data and methods of combining data from studies, including any planned exploration of consistency (such as I^2, Kendall's τ)	6
	15c	Describe any proposed additional analyses (such as sensitivity or subgroup analyses, meta-regression)	6
	15d	If quantitative synthesis is not appropriate, describe the type of summary planned	6
Meta-bias(es)	16	Specify any planned assessment of meta-bias(es) (such as publication bias across studies, selective reporting within studies)	6 + 7
Confidence in cumulative evidence	17	Describe how the strength of the body of evidence will be assessed (such as GRADE)	7

*** It is strongly recommended that this checklist be read in conjunction with the PRISMA-P Explanation and Elaboration (cite when available) for important clarification on the items. Amendments to a review protocol should be tracked and dated. The copyright for PRISMA-P (including checklist) is held by the PRISMA-P Group and is distributed under a Creative Commons Attribution Licence 4.0.**

From: Shamseer L, Moher D, Clarke M, Ghersi D, Liberati A, Petticrew M, Shekelle P, Stewart L, PRISMA-P Group. Preferred reporting items for systematic review and meta-analysis protocols (PRISMA-P) 2015: elaboration and explanation. BMJ. 2015 Jan 2;349(jan02 1):g7647.

For peer review only

BMJ Open

What are the prognostic factors for the development of incontinence-associated dermatitis (IAD): A protocol for a Systematic Review and Meta-Analysis

Journal:	*BMJ Open*
Manuscript ID	bmjopen-2023-073115.R1
Article Type:	Protocol
Date Submitted by the Author:	20-Apr-2023
Complete List of Authors:	Deprez, Julie; Örebro universitet, Swedish Centre for Skin and Wound Research (SCENTR), School of Health Sciences; Ghent University, Skin Integrity Research Group (SKINT), University Centre for Nursing and Midwifery, Department of Public Health and Primary Care<br>Kottner, Jan; Charite Universitatsmedizin Berlin, Institute for Clinical Nursing Science; Ghent University, Skin Integrity Research Group (SKINT), University Centre for Nursing and Midwifery, Department of Public Health and Primary Care<br>Eilegård Wallin, Alexandra; Örebro universitet, Swedish Centre for Skin and Wound Research (SCENTR), School of Health Sciences<br>Ohde, Nils; Charite Universitatsmedizin Berlin, Institute for Clinical Nursing Science<br>Bååth, Carina ; Karlstad University, Department of Health Sciences, Faculty of Health, Science and Technology; Østfold University College - Campus Frederikstad, Faculty of Health, Welfare and Organisation<br>Hommel, Ami ; Malmo University, Department of Care Science<br>Hultin, Lisa; Upsalla University , Department of Public Health and Caring Sciences; Upsalla University Hospital<br>Josefson, Anna; Örebro universitet, School of Medical Sciences, Faculty of Medicine and Health; Örebro University Hospital, Department of Dermatology<br>Beeckman, Dimitri; Örebro universitet, Swedish Centre for Skin and Wound Research (SCENTR), School of Health Sciences; Ghent University, Skin Integrity Research Group (SKINT), University Centre for Nursing and Midwifery, Department of Public Health and Primary Care
**Primary Subject Heading**:	Nursing
Secondary Subject Heading:	Dermatology, Nursing
Keywords:	Prognosis, Systematic Review, Risk Factors, WOUND MANAGEMENT

SCHOLARONE™
Manuscripts

TITLE PAGE

Article Title: *What are the prognostic factors for the development of incontinence-associated dermatitis (IAD): A protocol for a Systematic Review and Meta-Analysis*

Author Information:

DEPREZ Julie[1,2], KOTTNER Jan[2,3], EILEGÅRD WALLIN Alexandra[1], OHDE Nils[3], BÅÅTH Carina[4,5], HOMMEL, Ami[6], HULTIN Lisa[7,8], JOSEFSON Anna[9,10], BEECKMAN, Dimitri[1,2,11,12]

[1] Swedish Centre for Skin and Wound Research (SCENTR), Faculty of Health and Medicine, School of Health Sciences, Örebro University, Örebro, Sweden

[2] Skin Integrity Research Group (SKINT), University Centre for Nursing and Midwifery, Department of Public Health and Primary Care, Ghent University, Ghent, Belgium

[3] Institute for Clinical Nursing Science, Charité Universitätsmedizin Berlin, Berlin, Germany

[4] Department of Health Sciences, Faculty of Health, Science and Technology, Karlstad University, Karlstad, Sweden

[5] Faculty of Health, Welfare and Organisation, Østfold University College, Fredrikstad, Norway

[6] Department of Care Science Malmö University, Malmö University, Malmö, Sweden

[7] Department of Public Health and Caring Sciences, Uppsala University, Uppsala, Sweden

[8] Uppsala University Hospital, Uppsala, Sweden

[9] School of Medical Sciences, Faculty of Health and Medicine, Örebro University, Örebro, Sweden

[10] Department of Dermatology, University Hospital Örebro, Örebro, Sweden

[11] Research Unit of Plastic Surgery, Department of Clinical Research, Faculty of Health Sciences, Odense, Denmark

[12] School of Nursing & Midwifery, Royal College of Surgeons in Ireland (RCSI), Dublin, Ireland

CORRESPONDING AUTHOR

Julie DEPREZ
C. Heymanslaan 10, 9000 Ghent, Belgium
julie.deprez@oru.se

Word Count: 1.701

Keywords:
Prognostic factors, prognosis, risk factors, incontinence associated dermatitis, systematic review, meta-analysis.

For peer review only

ABSTRACT

Introduction

Incontinence associated dermatitis (IAD) is irritant contact dermatitis and skin damage associated with prolonged skin contact with urine and/or faeces. Identifying prognostic factors for the development of IAD may improve management, facilitate prevention, and inform future research.

Methods and analysis

This protocol follows the guidelines of the Preferred Reporting Items for Systematic Review and Meta-Analysis Protocols (PRISMA-P). Prospective and retrospective observational studies or clinical trials in which prognostic factors associated with the development of IAD are described are eligible. There are no restrictions on study setting, time, language, participant characteristics, or geographic regions. Reviews, editorials, commentaries, methodological articles, letters to the editor, cross-sectional and case-control studies, and case reports, are excluded. PubMed, CINAHL, EMBASE, and The Cochrane Library will be searched from inception until May 2023. Two independent reviewers will independently evaluate studies. The Quality in Prognostic Studies tool will be used to assess the risk of bias, and the CHARMS-PF checklist will be used for data extraction of the included studies. Separate analyses will be conducted for each identified prognostic factor, with adjusted and unadjusted estimated measures analysed separately. Evidence will be summarised with a meta-analysis when possible, and narratively otherwise. The Q and I2 statistics will be calculated in order to quantify heterogeneity. The quality of the evidence obtained will be evaluated according to the Grades of Recommendation Assessment, Development and Evaluation guidance.

Ethics and dissemination

No ethical approval is needed since all data is already publicly accessible. The results of this work will be published in a peer-reviewed scientific journal.

ARTICLE SUMMARY

STRENGTHS AND LIMITATIONS OF THIS STUDY

- This is the first systematic review to summarize the quality of evidence of prognostic factors for IAD.
- The risk of bias and the certainty of evidence will be evaluated using state-of-the-art methods for each prognostic factor separately.
- Because of interaction and confounding the generalisability of the identified prognostic factors might be limited.

For peer review only

MAIN TEXT

INTRODUCTION

Incontinence associated dermatitis (IAD) is one form of irritant contact dermatitis (EK02.22) and describes cutaneous inflammation and skin damage associated with prolonged skin contact with urine and/or faeces. (1, 2)

Clinical signs of IAD include skin erythema and oedema, occasionally with bullae and serous exudate, erosion, or subsequent cutaneous infection. (3, 4) Patients with IAD may experience discomfort, pain, burning, itching, or tingling. (5) The presence of IAD can result in an excessive burden of care, loss of independence, disruption in activities and/or sleep, and a reduction in quality of life, which worsens with higher frequency and quantity of soiling. (1, 6, 7) Additionally, IAD is associated with an increased risk of secondary infections, and the development of pressure ulcers. (1, 8, 9, 10) Managing all of these require additional resources, including prolonged hospital stays, resulting in a rise in healthcare expenses. (4, 11)

Prevalence ranges between 5.2% and 46%, depending on the type of setting and population under study. (9, 12)

By definition, incontinence is a prerequisite for having IAD, but in fact not all incontinent individuals develop IAD. (13) This suggests that many other factors and characteristics increase or decrease a person's susceptibility of developing IAD. Risk factors for IAD have been described (14, 15), but to our knowledge a systematic review and evidence appraisal on prognostic factors has not yet been conducted. 'A prognostic factor is any variable that is associated with the risk of a subsequent health outcome among people with a particular health condition.' (16, p. 2)

As such, our objective is to conduct a systematic review, and meta-analysis of prognostic factors associated with the development of IAD.

Review question

What are prognostic factors associated with the development of IAD in incontinent patients?

METHODS AND ANALYSIS

This protocol conforms to the Preferred Reporting Items for Systematic Reviews and Meta-Analyses Protocols (PRISMA-P) checklist. The protocol will be registered with the PROSPERO international prospective register of systematic reviews. The review is scheduled to be conducted between May 2023 and May 2024.

Eligibility criteria

Table 1 shows the description of the PICOTS (Population, Index prognostic factor, Comparator prognostic factor, Outcome, Timing, Setting) aspects. (16) Newborns, infants and children under 10 years of age will be excluded, due to differences in the structural and physiological properties of their skin compared to adults. For instance, the pH of neonatal and infant skin is typically higher, making them more susceptible to irritants and contributing to the development of conditions such as diaper

For peer review only

dermatitis. Additionally, the skin of new-borns is thinner and more vulnerable than that of adults. (17) The following study designs are eligible: RCTs, CCTs, prospective and retrospective cohort studies. Reviews, editorials, commentaries, methodological articles, letters to the editor, cross-sectional studies, case-control studies, and case reports, will be excluded. Case-control studies, although useful to identify prognostic factors, will be excluded due to the high risk of bias. (18) There are no restrictions on study setting, time, language, participant characteristics, or geographic regions. Studies will be rejected if it is not clear from the publication that the predictors existed prior to the onset of IAD. Due to the variety of language used to describe incontinence-associated skin problems (19, 20), all variations of terms will be included. If they describe the skin's reactive response to repeated exposure to urine and/or faeces, which may appear as inflammation and erythema with or without erosion or denudation. (3, 4, 21)

TABLE 1: Key items for framing aim, search strategy, and study inclusion and exclusion criteria for systematic review, following PICOTS

	**Definition**
**Population**	Incontinent adults (>18y) and adolescents (age 10 – 18y) (22)<br>Excluded: New borns, infants and young children (<10y)
**Index prognostic factor**	Any variable that existed prior to the onset of IAD and assessed for association with IAD
**Comparator prognostic factors**	Any other variable that existed before the onset of IAD
**Outcomes**	(1) Development of a new IAD<br>(2) Severity of IAD
**Time**	Minimum: 3 days<br>Maximum: the duration of the study or observation period
**Setting**	Any healthcare setting (Examples include but are not limited to: hospitals, nursing homes, community care…)

Information sources

To identify articles, the databases PubMed, CINAHL, EMBASE, and The Cochrane Library will be searched. Additional relevant studies will be identified by cited and citing references. ClinicalTrials.gov (www.clinicaltrials.gov) and the WHO International Clinical Trials Registry Platform (apps.who.int/trialsearch/) will be searched for unpublished and on-going studies. Articles will be electronically retrieved and in cases where electronic copies are unavailable, hard copies of these articles will be requested from the university library.

Search strategy

A library technician with expertise in systematic reviews at the Örebro University, Örebro, Sweden has been consulted to develop the search methods. The search strategy consists of key terms combined with Boolean operators. Key terms are defined using the PICOTS and include incontinence associated dermatitis and prognostic factors using various combinations and synonyms. The Boolean

For peer review only - http://bmjopen.bmj.com/site/about/guidelines.xhtml

operator OR is used to combine search terms for the same concept. The Boolean operator AND is used to combine the concepts. The search strategy will be adapted to each database. When appropriate MeSH terms or comparable alternatives will be used (see online supplementary appendix).

Study records

Data management

Retrieved records will be imported in Endnote and duplicates will be removed using the duplicate search function and by manually reviewing the list. Articles will then be imported into Covidence (23), an online tool that facilitates blind article screening by multiple reviewers.

Selection process

Titles and abstracts of all identified records will be reviewed for suitability by two researchers independently using the systematic review software Covidence (23). For each excluded article, reasons for exclusion will be specified. Disagreement or doubt will be resolved by consensus and if consensus cannot be reached, a full-text screening will be conducted, and a third reviewer will be consulted. The full texts of the remaining articles will be individually assessed for eligibility by the same reviewers. Any doubts will be resolved by a third reviewer.

Data collection process

The data extraction will be independently completed by two reviewers. Data extraction forms will be used to extract the relevant information and evidence. Any disagreement will be resolved through consultation with another reviewer.

Data items

The Checklist for Critical Appraisal and Data Extraction for Systematic Reviews of Prediction Modelling Studies – Prognostic Factors (CHARMS-PF) (16) will guide data extraction. It has nine domains that cover information required to extract data in a reliable manner: source of data, participants, outcomes to be predicted, prognostic factors, sample size, missing data, analysis, results and interpretation and discussion. Table 2 shows a list of data items that will be extracted.

TABLE 2: Data charting variables and domains relating to article description

Domain	Selected items and examples
**General study information**	Title, authors names, year of publication
**Study characteristics**	Sample size, study design, duration of follow-up, geographical location
**Participant characteristics**	Participant description, eligibility criteria
**Setting**	Type of care institution (hospital (unit), homecare, residential care…)
**Definition of outcomes**	IAD definition used by authors.<br>Incontinence definition used by authors.
**Prognostic factors**	Examples include but are not limited to: presence of faecal incontinence and/or urinary incontinence, care dependency, level of personal hygiene…

For peer review only - http://bmjopen.bmj.com/site/about/guidelines.xhtml

**Missing data**	Attrition (quantification and reasons), handling of missing data by the study authors
**Analysis**	Univariate analysis/logistic regression/Cox regression/other
**Results**	*Estimates reported between the prognostic factor and each review outcome*: (a) unadjusted estimate (b) adjusted estimates.<br>*Type of measure of association*: odds ratio (OR), risk ratio (RR) or hazard ratio (HR)

Outcomes and prioritization

The main predicted outcome will be the development of new IAD, defined as an irritant contact dermatitis resulting from prolonged contact of the skin with urine and/or faeces. (2) The severity of IAD will also be examined.

Assessment of a risk of bias in individual studies

The Quality in Prognostic Factor Studies (QUIPS) (24) tool will be used to assess the risk of bias of each of the prognostic factors in individual studies. It has six domains (study participants, study attrition, prognostic factor measurement, outcome measurement, study confounding and statistical analysis and reporting) with a series of prompting items for each domain. Each domain is assigned a risk of bias rating of high, moderate, or low, and the overall risk of bias of a study is determined by a cumulative rating of all domains. The risk of bias will be independently assessed by two reviewers. Disagreements will be discussed with a third author until consensus is reached.

Data synthesis

A table will be created summarizing the characteristics of identified prognostic factors. Then, the strength and directions of association will be summarized for each prognostic factor separately. ORs, RRs, and HRs will be used as measures of association. Mean differences will be considered for continuous prognostic factors. Adjusted and unadjusted estimates for each prognostic factor will be analysed separately. When at least five studies report similar measures of association, a meta-analysis will be conducted for each prognostic factor, if the measurement of this factor is comparable. (16, 25) When neither OR, RR, or HR nor corresponding SEs are reported, any available data from the included study, such as confidence intervals, Kaplan-Meier curves, logrank test p values, will be utilized to estimate the associations if possible. (26, 27) A random-effects model will be used when conducting meta-analysis, since it is assumed that there is a high degree of variability among studies due to clinical and methodological differences. (28) The effects of each prognostic factor will be estimated as an OR or standardised mean difference with a 95% CI and T2, which indicates between-study variance. Furthermore, a 95% prediction interval will also be calculated. (29) The heterogeneity between studies in each meta-analysis will be assessed visually by examining forest plots and calculating the Q and I2 statistics. (30) When it is not feasible to conduct a quantitative synthesis, the evidence will be summarised narratively.

Bias

The presence of small study bias, including publication bias, will be evaluated graphically by examining the presence of asymmetry in a funnel plots. Additionally, the Egger's test will be used to make a

For peer review only - http://bmjopen.bmj.com/site/about/guidelines.xhtml

statistical assessment if a meta-analysis is based on 10 or more studies for an outcome. (31) For this test, the natural logarithmic scale of the OR must be regressed against its SE, and statistical significance for asymmetry is set at the 10% level due to the low power of the test. If publication bias is statistically suspected, the method of trim and fill can be used to estimate the number of missing studies and provide an adjusted summary effect. (32) To assess selective reporting, the consistency of study findings with its protocol must be examined, if available.

Confidence in cumulative evidence

Using the modified GRADE approach, the quality of evidence for each prognostic factor will be evaluated. The certainty rating for observational studies will begin at high, while the secondary analysis of highly controlled RCTs will start at low. The domains of risk of bias, inconsistency, indirectness, imprecision, publication bias, large effect, dose response, and plausible confounding will be taken into account when deciding to downgrade or upgrade the evidence's certainty. (33)

Patient and public involvement

No patients or service users were involved in the design of this review protocol.

ETHICS AND DISSEMINATION

This systematic review does not require ethics approval as it will be a summary of previously published evidence. No individual patient data will be obtained or accessed. Therefore, there is no concerning ethical issue in the conduct of this research. Following the completion of the systematic review, findings will be presented to academic audiences at international conferences focusing on skin integrity. The results will also be published in a peer-reviewed academic journal to reach clinical and academic experts interested in the topic. Plain language summaries and presentations to hospitals and other relevant clinical programmes will also be developed.

For peer review only

FOOTNOTES

Author Contributions:

JK, AEW, DB, CB, LH, AJ and AH provided the idea of the topic. All authors contributed to the conception of the research question. JD designed and wrote the protocol in close collaboration with JK, AEW, NO and DB. All authors contributed to the development of search strategies, eligibility criteria, and methodology for data synthesis. All authors revised the protocol critically for important intellectual content. All authors contributed and provided feedback to the draft protocol and approved the final version of this protocol. JD and NO will review the titles and abstracts of all studies obtained using the search strategy to exclude articles that do not meet the eligibility criteria.

Conflict of interest statement:

The authors declare that there is no conflict of interest.

Acknowledgments:

We acknowledge the support and funding from Vetenskapsrådet.

Funding Statement:

This work was supported by Vetenskapsrådet, grant number 2021-02653.

Data Availability Statement:

Data sharing not applicable as no datasets are generated and/or analysed for this study.

For peer review only

REFERENCES

1. Beeckman D, Campbell J, Campbell K, et al. Proceedings of the Global IAD Expert Panel. Incontinence associated dermatitis: moving prevention forward. Wounds International. 2015.
2. World Health Organization (WHO). International classification of diseases for mortality and morbidity statistics (11th Revision). 2018. Available from: https://icd.who.int/browse11/l-m/en [Accessed 9 Jan 2023].
3. Beeckman D, Van Damme N, Schoonhoven L, et al. Interventions for preventing and treating incontinence-associated dermatitis in adults. Cochrane Database Syst Rev. 2016;11(11):Cd011627.
4. Raepsaet C, Fourie A, Van Hecke A, et al. Management of incontinence-associated dermatitis: A systematic review of monetary data. International wound journal. 2021;18(1):79-94.
5. Van den Bussche K, Kottner J, Beele H, et al. Core outcome domains in incontinence-associated dermatitis research. Journal of advanced nursing. 2018;74(7):1605-17.
6. Bartlett L, Nowak M, Ho YH. Impact of fecal incontinence on quality of life. World J Gastroenterol. 2009;15(26):3276-82.
7. Minassian VA, Devore E, Hagan K, et al. Severity of urinary incontinence and effect on quality of life in women by incontinence type. Obstetrics and gynecology. 2013;121(5):1083-90.
8. Demarre L, Verhaeghe S, Van Hecke A, et al. Factors predicting the development of pressure ulcers in an at-risk population who receive standardized preventive care: secondary analyses of a multicentre randomised controlled trial. Journal of advanced nursing. 2015;71(2):391-403.
9. Gray M, Giuliano KK. Incontinence-Associated Dermatitis, Characteristics and Relationship to Pressure Injury: A Multisite Epidemiologic Analysis. J Wound Ostomy Continence Nurs. 2018;45(1):63-7.
10. Van Damme N, Vanryckeghem E, Verhaeghe S, et al. Incontinence-associated dermatitis in elderly : a qualitative phenomenological study on patient experiences. 18th Annual meeting of the European Pressure Ulcer Advisory Panel (EPUAP 2015); Ghent, Belgium. European Pressure Ulcer Advisory Panel, 18th Annual meeting, Abstracts: EPUAP Business Office; 2015. p. 65 -
11. Cunich M, Barakat-Johnson M, Lai M, et al. The costs, health outcomes and cost-effectiveness of interventions for the prevention and treatment of incontinence-associated dermatitis: A systematic review. Int J Nurs Stud. 2022;129:104216.
12. Babino G, Argenziano G. Incontinence-Associated Dermatitis: An Insidious and Painful Condition. In: Docimo L, Brusciano L, editors. Anal Incontinence: Clinical Management and Surgical Techniques. Cham: Springer International Publishing; 2023. p. 179-87.
13. Van Damme N, Clays E, Verhaeghe S, et al. Independent risk factors for the development of incontinence-associated dermatitis (category 2) in critically ill patients with fecal incontinence: A cross-sectional observational study in 48 ICU units. Int J Nurs Stud. 2018;81:30-9.
14. Heywood N, Holloway S. Risk factors for incontinence associated dermatitis: an evidence-based review. Wounds UK. 2014;10(4):22 - 33.
15. Kottner J, Blume-Peytavi U, Lohrmann C, et al. Associations between individual characteristics and incontinence-associated dermatitis: a secondary data analysis of a multi-centre prevalence study. Int J Nurs Stud. 2014;51(10):1373-80.

For peer review only

16. Riley RD, Moons KGM, Snell KIE, et al. A guide to systematic review and meta-analysis of prognostic factor studies. BMJ. 2019;364:k4597.
17. Oranges T, Dini V, Romanelli M. Skin Physiology of the Neonate and Infant: Clinical Implications. Advances in Wound Care. 2015;4(10):587-95.
18. Foroutan F, Guyatt G, Zuk V, et al. GRADE Guidelines 28: Use of GRADE for the assessment of evidence about prognostic factors: rating certainty in identification of groups of patients with different absolute risks. Journal of Clinical Epidemiology. 2020;121:62-70.
19. Beeckman D, Schoonhoven L, Verhaeghe S, et al. Prevention and treatment of incontinence-associated dermatitis: literature review. J Adv Nurs. 2009;65(6):1141-54.
20. Dunk AM, Broom M, Kottner J, et al. Is it time to reconsider the terminology for diaper dermatitis? J Tissue Viability. 2023.
21. Gray M, Bohacek L, Weir D, et al. Moisture vs pressure: making sense out of perineal wounds. J Wound Ostomy Continence Nurs. 2007;34(2):134-42.
22. World Health Organization (WHO). Adolescents Health. Available from: https://www.who.int/health-topics/adolescent-health#tab=tab_1 [Accessed 25 Jan 2023].
23. Veritas Health Innovation. Covidence systematic review software. 2023.
24. Hayden JA, van der Windt DA, Cartwright JL, et al. Assessing bias in studies of prognostic factors. Ann Intern Med. 2013;158(4):280-6.
25. Jackson D, Turner R. Power analysis for random-effects meta-analysis. Res Synth Methods. 2017;8(3):290-302.
26. Parmar MK, Torri V, Stewart L. Extracting summary statistics to perform meta-analyses of the published literature for survival endpoints. Stat Med. 1998;17(24):2815-34.
27. Tierney JF, Stewart LA, Ghersi D, et al. Practical methods for incorporating summary time-to-event data into meta-analysis. Trials. 2007;8:16.
28. DerSimonian R, Laird N. Meta-analysis in clinical trials. Control Clin Trials. 1986;7(3):177-88.
29. Riley RD, Higgins JPT, Deeks JJ. Interpretation of random effects meta-analyses. BMJ. 2011;342:d549.
30. Higgins JPT, Thompson SG, Deeks JJ, et al. Measuring inconsistency in meta-analyses. BMJ. 2003;327(7414):557-60.
31. Egger M, Smith GD, Schneider M, et al. Bias in meta-analysis detected by a simple, graphical test. BMJ. 1997;315(7109):629-34.
32. Duval S, Tweedie R. Trim and fill: A simple funnel-plot-based method of testing and adjusting for publication bias in meta-analysis. Biometrics. 2000;56(2):455-63.
33. Foroutan F, Guyatt G, Zuk V, et al. GRADE Guidelines 28: Use of GRADE for the assessment of evidence about prognostic factors: rating certainty in identification of groups of patients with different absolute risks. J Clin Epidemiol. 2020;121:62-70.

For peer review only - http://bmjopen.bmj.com/site/about/guidelines.xhtml

Field codes:
/= MeSH term
exp= exploded MeSH term
ab.= abstract
kf.= keyword heading word
ti.= title
tw.= title/abstract

Database(s): **Ovid MEDLINE(R) ALL** 1946 to April 13, 2023
Search Strategy:

#	Searches
**Incontinence**	
1	exp Urinary Incontinence/
2	Incontinence Pads/
3	Absorbent Pads/
4	Diapers, Adult/
5	Fecal Incontinence/
6	(incontinen* or diaper* or napkin* or nappy or perineal or perineum or "absorbent pads" or underpad*).ab,kf,ti.
7	1 or 2 or 3 or 4 or 5 or 6
**Dermatis/skin problems**	
8	Dermatitis/ or exp Dermatitis, Contact/ or exp Intertrigo/ or exp Dermatitis, Irritant/ or "Erythema"/
9	(dermati* or erythema or rash* or intertrig* or erythema or "moisture associated skin damage" or MASD or "skin barrier*" or "skin breakdown" or "skin condition" or "skin damage" or "skin health" or "fragile skin" or "heathy skin" or "skin problem*" or erythema or "perianal skin" or "Buttock skin" or "wetness lesion*" or "moisture lesion*").ab,kf,ti.
10	8 or 9
**Prognostic factors**	
11	exp Risk/
12	risk.tw.
13	exp Cohort Studies/
14	cohort.tw.
15	exp Prognosis/
16	"prognos*".tw.
17	"predict*".tw.
18	exp Incidence/
19	incidence.tw.
20	"causal factor".tw.

21	course.tw.
22	exp Follow-Up Studies/
23	(prognos* or predict* or risk or incidence or indicat* or factor* or predisposition or predisposed or etiology or aetiology).ab,kf,ti.
24	11 or 12 or 13 or 14 or 15 or 16 or 17 or 18 or 19 or 20 or 21 or 22 or 23
**Sets combined**	
25	7 and 10 and 24

Published search filters for prognosis/prognostic factors were consulted in the development of the search strategy. Line 11-21 can be found in the filter by Stallings et al.(1). Line 16, 17 and 21 are also used I by Kavanagh et al.(2). Both filters also use additional terms associated with mortality, but those terms were not used since IAD is not mortal. Because articles on prognostic factors may still use a terms not present in the filters, line 23 also includes some additional terms.

1. Stallings E, Gaetano-Gil A, Alvarez-Diaz N, Sola I, Lopez-Alcalde J, Molano D, et al. Development and evaluation of a search filter to identify prognostic factor studies in Ovid MEDLINE. Bmc Med Res Methodol. 2022;22:107. DOI: 10.1186/s12874-022-01595-9

2. Kavanagh PL, Frater F, Navarro T, LaVita P, Parrish R, Iorio A. Optimizing a literature surveillance strategy to retrieve sound overall prognosis and risk assessment model papers. J Am Med Inform Assoc JAMIA. 2021;28:766–71.

For peer review only

PRISMA-P (Preferred Reporting Items for Systematic review and Meta-Analysis Protocols) 2015 checklist: recommended items to address in a systematic review protocol*

Section and topic	Item No	Checklist item	Reported on page #
**ADMINISTRATIVE INFORMATION**			
Title:			
Identification	1a	Identify the report as a protocol of a systematic review	1
Update	1b	If the protocol is for an update of a previous systematic review, identify as such	n/a
Registration	2	If registered, provide the name of the registry (such as PROSPERO) and registration number	n/a
Authors:			
Contact	3a	Provide name, institutional affiliation, e-mail address of all protocol authors; provide physical mailing address of corresponding author	1
Contributions	3b	Describe contributions of protocol authors and identify the guarantor of the review	8
Amendments	4	If the protocol represents an amendment of a previously completed or published protocol, identify as such and list changes; otherwise, state plan for documenting important protocol amendments	n/a
Support:			
Sources	5a	Indicate sources of financial or other support for the review	8
Sponsor	5b	Provide name for the review funder and/or sponsor	8
Role of sponsor or funder	5c	Describe roles of funder(s), sponsor(s), and/or institution(s), if any, in developing the protocol	n/a
**INTRODUCTION**			
Rationale	6	Describe the rationale for the review in the context of what is already known	3
Objectives	7	Provide an explicit statement of the question(s) the review will address with reference to participants, interventions, comparators, and outcomes (PICO)	3
**METHODS**			
Eligibility criteria	8	Specify the study characteristics (such as PICO, study design, setting, time frame) and report characteristics (such as years considered, language, publication status) to be used as criteria for eligibility for the review	3 + 4
Information sources	9	Describe all intended information sources (such as electronic databases, contact with study authors, trial registers or other grey literature sources) with planned dates of coverage	4
Search strategy	10	Present draft of search strategy to be used for at least one electronic database, including planned limits, such that it could be repeated	Supplementary file

For peer review only

Study records:			
Data management	11a	Describe the mechanism(s) that will be used to manage records and data throughout the review	5
Selection process	11b	State the process that will be used for selecting studies (such as two independent reviewers) through each phase of the review (that is, screening, eligibility and inclusion in meta-analysis)	5
Data collection process	11c	Describe planned method of extracting data from reports (such as piloting forms, done independently, in duplicate), any processes for obtaining and confirming data from investigators	5
Data items	12	List and define all variables for which data will be sought (such as PICO items, funding sources), any pre-planned data assumptions and simplifications	5 + 6
Outcomes and prioritization	13	List and define all outcomes for which data will be sought, including prioritization of main and additional outcomes, with rationale	6
Risk of bias in individual studies	14	Describe anticipated methods for assessing risk of bias of individual studies, including whether this will be done at the outcome or study level, or both; state how this information will be used in data synthesis	6
Data synthesis	15a	Describe criteria under which study data will be quantitatively synthesised	6
	15b	If data are appropriate for quantitative synthesis, describe planned summary measures, methods of handling data and methods of combining data from studies, including any planned exploration of consistency (such as I^2, Kendall's τ)	6
	15c	Describe any proposed additional analyses (such as sensitivity or subgroup analyses, meta-regression)	6
	15d	If quantitative synthesis is not appropriate, describe the type of summary planned	6
Meta-bias(es)	16	Specify any planned assessment of meta-bias(es) (such as publication bias across studies, selective reporting within studies)	6 + 7
Confidence in cumulative evidence	17	Describe how the strength of the body of evidence will be assessed (such as GRADE)	7

*** It is strongly recommended that this checklist be read in conjunction with the PRISMA-P Explanation and Elaboration (cite when available) for important clarification on the items. Amendments to a review protocol should be tracked and dated. The copyright for PRISMA-P (including checklist) is held by the PRISMA-P Group and is distributed under a Creative Commons Attribution Licence 4.0.**

From: Shamseer L, Moher D, Clarke M, Ghersi D, Liberati A, Petticrew M, Shekelle P, Stewart L, PRISMA-P Group. Preferred reporting items for systematic review and meta-analysis protocols (PRISMA-P) 2015: elaboration and explanation. BMJ. 2015 Jan 2;349(jan02 1):g7647.

For peer review only
